# Supplementary material for: Comparative Study of Selected Excipients’ Influence on Carvedilol Release from Hypromellose Matrix Tablets
Source: Pharmaceutics. 2023 May 18;15(5):1525. doi: 10.3390/pharmaceutics15051525 (PMC10223957; doi:10.3390/pharmaceutics15051525)
Supplement: Supplementary file 1 [file pharmaceutics-15-01525-s001.zip › pharmaceutics-2359498-supplementary.pdf]

pharmaceutics MDPI

Table S1. Experimental results of carvedilol release. Data is shown for PEG/PEO, PVP and mannitol groups. The results given are mean values with corresponding standard deviations shown in parentheses (n = 4).

t [min]	Polyglykol® 4000 P	Polyglykol® 8000 P	POLYOX™ WSR N-80 (LEO NF Grade)	KOLLIDON® 25	KOLLIDON® 90 F	C*Pharm Mannidex 16700	PEARLITOL® 160C	Parteck® M 100	Parteck® M 200
10	17.1 (0.7)	12.4 (1.16)	1.9 (0.38)	12.2 (2.44)	1.3 (0.34)	9.8 (1.47)	10.5 (1)	5.3 (0.65)	6.5 (0.41)
20	33.7 (2.8)	30 (1.57)	5.7 (1.01)	25 (4.98)	3.2 (0.79)	15.6 (1.93)	16.5 (1.12)	17.4 (2.06)	17.3 (3.04)
30	46.9 (4.11)	45.4 (1.9)	10.5 (1.73)	35.9 (7.05)	5.3 (1.39)	20.6 (2.37)	21.6 (1.2)	35.3 (12.06)	30.9 (11.31)
45	63.7 (6.87)	64.1 (2.01)	18.7 (2.28)	49.7 (12.58)	8.5 (2.23)	33.6 (15.08)	28.7 (1.94)	55.3 (16.19)	48 (16.88)
60	76.1 (8.82)	77.8 (1.62)	27.2 (1.99)	60 (19.45)	11.9 (3.15)	40.3 (17.66)	37.4 (6.6)	70.1 (12.52)	62.8 (16.02)
90	89.8 (7.8)	94.2 (1.28)	42.8 (1.55)	72.1 (25.81)	19.1 (4.88)	50.2 (17.3)	54.5 (15.89)	86.3 (8.59)	79.8 (14.76)
120	91.8 (5.46)	96.4 (2.11)	56.4 (2.47)	76.2 (23.86)	26.5 (6.25)	59.9 (16.42)	66.5 (17.39)	93.1 (6.6)	87 (11.68)
150	92.8 (4.33)	97 (1.4)	67.9 (3.5)	79.2 (21.53)	34.1 (7.28)	69.6 (15.65)	75.4 (16.14)	97.1 (4.03)	91.7 (8.28)
180	93.6 (3.32)	97.2 (1.38)	77.2 (4.3)	82.6 (18.62)	41.3 (7.63)	77.1 (14.25)	82.1 (14.36)	99.3 (2.55)	94.7 (5.75)
210	94.3 (2.48)	97.1 (1.37)	84.8 (4.46)	85.4 (16.18)	48.7 (7.33)	83.7 (13.35)	87.4 (12.67)	101.2 (1.25)	97.6 (3.3)
240	94.9 (1.81)	97.2 (1.36)	91 (3.34)	88.2 (14.23)	55.5 (7.1)	88.2 (10.12)	91.9 (10.93)	102.1 (0.69)	99 (1.19)
270	95.5 (1.19)	97.2 (1.34)	95.4 (2.2)	90.6 (12.26)	61.7 (6.85)	91.8 (7.64)	95.1 (8.8)	102.1 (0.75)	99.7 (0.53)
300	95.8 (0.94)	97.1 (1.37)	97.2 (0.73)	92.5 (10.26)	67.2 (6.68)	94.6 (5.62)	97.5 (6.99)	102.1 (0.75)	99.8 (0.63)
330	96.2 (0.84)	97.2 (1.36)	97.9 (0.84)	94.3 (8.37)	72.4 (6.74)	97.2 (4.31)	99.3 (5.55)	102.2 (0.83)	99.9 (0.67)
360	96.3 (0.91)	97.1 (1.35)	98.1 (1.2)	95.8 (6.73)	77 (6.86)	98.7 (3.11)	100.5 (4.37)	102.1 (0.86)	100 (0.65)
420	96.3 (1.02)	97.2 (1.31)	98.1 (1.23)	98.2 (4.3)	85.1 (6.46)	100.7 (0.9)	102.9 (1.99)	102.2 (0.94)	100 (0.69)
480	96.3 (1.02)	97.1 (1.29)	98 (1.25)	100.1 (3.01)	91.3 (5.31)	101 (0.47)	103.7 (1.22)	102.2 (0.99)	100.1 (0.66)
540	96.3 (1.07)	97.1 (1.25)	98 (1.26)	101.3 (2.52)	95.9 (3.7)	101 (0.47)	104 (1.17)	102.2 (1.03)	100 (0.7)
600	96.3 (1.1)	97.1 (1.19)	98 (1.3)	102.1 (2.62)	98 (1.92)	100.8 (0.46)	104.2 (1.2)	102.2 (1.06)	100.1 (0.66)
660	96.3 (1.16)	97.1 (1.17)	97.9 (1.28)	102.4 (2.55)	99 (1.5)	100.8 (0.41)	104.4 (1.17)	102.3 (1.14)	100.1 (0.69)
720	96.1 (1.18)	97 (1.18)	97.9 (1.33)	102.5 (2.6)	99.5 (2.17)	100.8 (0.42)	104.5 (1.19)	102.2 (1.26)	100.1 (0.66)
780	96.1 (1.24)	97 (1.16)	97.9 (1.37)	102.6 (2.62)	99.6 (2.19)	100.6 (0.44)	104.7 (1.22)	102.2 (1.31)	100.2 (0.67)
840	96.1 (1.26)	96.9 (1.14)	97.8 (1.39)	102.7 (2.62)	99.6 (2.21)	100.6 (0.41)	104.9 (1.27)	102.1 (1.38)	100.2 (0.68)
900	96 (1.28)	96.9 (1.07)	97.7 (1.43)	102.9 (2.73)	99.6 (2.21)	100.6 (0.39)	105.1 (1.28)	102.1 (1.43)	100.1 (0.69)

960	96 (1.29)	96.9 (1.09)	97.7 (1.44)	102.9 (2.78)	99.6 (2.22)	100.5 (0.44)	105.2 (1.32)	102.1 (1.46)	100.1 (0.69)
1020	96 (1.34)	97 (1.19)	97.9 (1.28)	103 (2.8)	99.7 (2.22)	100.9 (0.63)	105.4 (1.31)	102.1 (1.44)	100.2 (0.66)
1080	95.9 (1.4)	97 (1.15)	97.8 (1.37)	103.2 (2.85)	99.7 (2.24)	100.9 (0.69)	105.7 (1.34)	102.1 (1.33)	100.2 (0.63)
1140	95.8 (1.45)	97 (1.19)	97.9 (1.31)	103.3 (2.87)	99.7 (2.21)	100.7 (0.37)	105.7 (1.41)	102.1 (1.45)	100.2 (0.69)
1200	95.8 (1.51)	97 (1.12)	97.9 (1.28)	103.4 (2.93)	99.7 (2.22)	100.9 (0.6)	106 (1.45)	102.1 (1.3)	100.3 (0.69)
1260	95.7 (1.49)	96.9 (1.12)	97.8 (1.44)	103.5 (2.91)	99.7 (2.22)	100.7 (0.51)	106.1 (1.49)	102.1 (1.46)	100.3 (0.69)
1320	95.7 (1.58)	97 (0.97)	98 (1.11)	103.3 (2.85)	99.7 (2.23)	100.8 (0.44)	106 (1.41)	102.1 (1.35)	100.3 (0.72)
1380	95.8 (1.51)	97 (1.17)	98 (1.29)	103.4 (3)	99.7 (2.3)	100.7 (0.37)	106 (1.53)	102.1 (1.44)	100.3 (0.7)
1440	95.7 (1.57)	97 (0.96)	97.9 (1.4)	103.4 (2.82)	99.8 (2.33)	101 (0.58)	106 (1.36)	102.2 (1.3)	100.2 (0.71)

Table S2. Experimental results of carvedilol release. Data is shown for the lactose group, sucrose and maltodextrin. The results given are mean values with corresponding standard deviations shown in parentheses (n = 4).

t [min]	Lactochem® Fine Powder	Lactochem® Crystals	SuperTab® 11SD	FlowLac® 100	Tablettose® 70	Granulated sugar N°1 600	GLUCIDEX® 19
10	7.2 (0.89)	21.5 (2.61)	30.1 (4.67)	8.7 (1.77)	27.1 (2.62)	3.3 (0.3)	3.7 (0.71)
20	10.4 (0.92)	26.5 (2.33)	34.4 (4.84)	11.8 (1.96)	32 (2.15)	5.4 (0.39)	5.6 (0.78)
30	13 (0.94)	30 (2.26)	37.2 (4.91)	14 (2.06)	35.6 (2.03)	7.2 (0.43)	7.2 (0.84)
45	16.6 (0.99)	34 (2.31)	40.6 (4.91)	16.9 (2.22)	39.8 (1.77)	9.6 (0.53)	9.2 (0.87)
60	19.7 (1.18)	37.3 (2.41)	43.6 (4.87)	19.5 (2.32)	43.1 (1.54)	12 (0.57)	11.3 (0.88)
90	25.1 (1.5)	43 (2.43)	48.8 (4.44)	24.1 (2.38)	48.8 (1.16)	16.4 (0.78)	15.4 (0.98)
120	29.7 (1.73)	47.9 (2.37)	53.4 (3.92)	28.3 (2.35)	53.4 (0.84)	20.7 (0.93)	19.2 (1.08)
150	33.9 (1.96)	52.2 (2.3)	57.2 (3.76)	32.2 (2.27)	57.4 (0.57)	24.8 (1.08)	22.8 (1.16)
180	37.9 (2.12)	56.1 (2.29)	60.6 (3.75)	36 (2.25)	61 (0.69)	28.7 (1.16)	26.3 (1.26)
210	41.7 (2.28)	59.7 (2.33)	63.7 (3.71)	39.7 (2.25)	64.2 (0.67)	32.3 (1.23)	29.9 (1.34)
240	45.5 (2.29)	63 (2.28)	66.6 (3.54)	43.1 (2.25)	67.2 (0.75)	35.9 (1.35)	33.3 (1.41)
270	49.1 (2.15)	66.1 (2.35)	69.3 (3.39)	46.6 (2.28)	70 (0.79)	39.3 (1.43)	36.8 (1.47)
300	52.6 (2.11)	69.1 (2.38)	72 (3.23)	50 (2.27)	72.7 (0.87)	42.7 (1.52)	40.1 (1.49)
330	55.9 (1.99)	71.9 (2.37)	74.5 (3.04)	53.1 (2.19)	75.4 (0.94)	45.9 (1.6)	43.4 (1.54)
360	59.1 (1.94)	74.6 (2.48)	77 (2.81)	56.3 (2.22)	77.9 (0.94)	49 (1.68)	46.7 (1.61)
420	65.1 (2.03)	79.7 (2.51)	81.6 (2.9)	62.3 (2.31)	82.5 (0.7)	54.8 (1.88)	52.9 (1.68)
480	70.7 (2.08)	84.4 (2.56)	86 (3.01)	68 (2.23)	87.5 (2.24)	60.3 (2)	60.8 (5.24)
540	76.3 (2.27)	88.5 (2.44)	90.7 (4.05)	73.3 (2.13)	92.7 (3.32)	65.3 (2.2)	67.1 (5.03)
600	84.3 (5.79)	93.2 (2.61)	94.7 (3.51)	80.2 (4.88)	95.6 (2.39)	70 (2.36)	72.7 (4.91)
660	90.8 (8.07)	98.6 (2.96)	97.7 (1.93)	86.9 (5.49)	97 (1.61)	74.2 (2.55)	77.6 (4.75)
720	94.8 (7.39)	101.4 (1.23)	99.5 (0.61)	93 (3.34)	97.3 (1.32)	77.9 (2.68)	81.8 (4.63)
780	98 (4.96)	101.8 (1.2)	100 (0.34)	97.2 (1.89)	97.2 (1.37)	81.2 (2.72)	85.4 (4.49)
840	100.3 (3.28)	101.9 (1.24)	100.1 (0.25)	98.9 (1.43)	97.2 (1.37)	84 (2.7)	88.4 (4.35)
900	101.8 (1.87)	101.8 (1.27)	100 (0.24)	99.6 (1.27)	97.2 (1.45)	86.2 (2.62)	91.1 (4.2)
960	102.7 (1.58)	101.8 (1.27)	100 (0.24)	99.8 (1.26)	97.1 (1.45)	88.1 (2.71)	93.3 (4.04)
1020	103.1 (1.9)	101.7 (1.36)	100 (0.34)	99.8 (1.3)	97.2 (1.46)	89.7 (2.79)	95.2 (3.82)

1080	103.2 (1.99)	101.7 (1.41)	99.9 (0.37)	99.7 (1.27)	97.3 (1.33)	90.8 (2.74)	96.7 (3.51)
1140	103.2 (2.01)	101.7 (1.45)	99.9 (0.41)	99.7 (1.29)	97.2 (1.37)	91.7 (2.61)	98 (3.17)
1200	103.2 (2.01)	101.7 (1.52)	99.9 (0.41)	99.7 (1.36)	97.4 (1.33)	92.3 (2.43)	99 (2.75)
1260	103.2 (2.03)	101.6 (1.56)	99.8 (0.42)	99.6 (1.35)	97.2 (1.42)	92.8 (2.32)	99.9 (2.33)
1320	103.1 (2.05)	101.6 (1.63)	99.6 (0.49)	99.4 (1.42)	97.3 (1.3)	93.1 (2.3)	100.5 (1.9)
1380	103.2 (2.06)	101.6 (1.62)	99.6 (0.5)	99.4 (1.42)	97.3 (1.36)	93.4 (2.22)	100.9 (1.67)
1440	103.4 (2.08)	101.7 (1.69)	99.6 (0.48)	99.3 (1.47)	97.1 (1.44)	93.5 (2.25)	101.1 (1.56)

Table S3. Experimental results of carvedilol release. Data is shown for DCP, pregelatinized starch, MCC and EC. The results given are mean values with corresponding standard deviations shown in parentheses (n = 4).

t [min]	DI-CAFOS® A12	EMCOMPRESS ® Anhydrous	STARCH 1500® ↓PS	STARCH 1500® ↑PS	AVICEL® PH-102	AVICEL® PH-200	ETHOCEL™ Standard 20 Premium
10	3.8 (0.12)	7 (0.41)	9.7 (2.34)	5.9 (0.42)	2 (0.74)	2.3 (0.19)	1.9 (0.17)
20	5.7 (0.15)	9.4 (0.54)	12 (2.89)	7.6 (0.5)	3.7 (0.76)	4 (0.38)	3.5 (0.25)
30	7.1 (0.24)	11.1 (0.57)	13.4 (2.9)	8.9 (0.54)	4.9 (0.88)	5.3 (0.54)	4.8 (0.29)
45	9.2 (0.33)	13.2 (0.6)	15.1 (2.96)	10.6 (0.57)	6.7 (0.96)	7.1 (0.66)	6.4 (0.42)
60	10.9 (0.44)	15 (0.65)	16.6 (2.93)	12 (0.62)	8.1 (1.08)	8.8 (0.79)	7.8 (0.46)
90	14.2 (0.57)	18.1 (0.78)	19.1 (2.9)	14.6 (0.66)	10.8 (1.17)	11.8 (0.88)	10.6 (0.61)
120	17.1 (0.74)	20.9 (1)	21.3 (2.99)	16.8 (0.72)	13.2 (1.18)	14.5 (0.88)	13.3 (0.73)
150	19.9 (0.89)	23.5 (1.28)	23.4 (2.93)	18.8 (0.75)	15.4 (1.21)	17 (0.88)	15.8 (0.75)
180	22.6 (1.1)	25.9 (1.48)	25.1 (2.92)	20.7 (0.83)	17.5 (1.21)	19.3 (0.88)	18.3 (0.84)
210	25.1 (1.27)	28.2 (1.71)	26.9 (2.93)	22.5 (0.93)	19.4 (1.17)	21.6 (0.85)	20.7 (0.85)
240	27.7 (1.47)	30.5 (1.96)	28.6 (2.87)	24.2 (1.09)	21.3 (1.17)	23.8 (0.86)	23 (0.83)
270	30 (1.63)	32.7 (2.22)	30.1 (2.91)	25.8 (1.2)	23.1 (1.16)	25.9 (0.86)	25.3 (0.86)
300	32.4 (1.79)	34.8 (2.44)	31.8 (2.95)	27.3 (1.3)	24.9 (1.21)	28 (0.86)	27.5 (0.85)
330	34.7 (1.94)	36.9 (2.67)	33.3 (2.92)	28.8 (1.41)	26.8 (1.21)	29.9 (0.91)	29.7 (0.83)
360	37 (2.14)	38.9 (2.9)	34.7 (2.98)	30.4 (1.52)	28.5 (1.19)	31.9 (0.93)	31.8 (0.79)
420	41.4 (2.42)	42.8 (3.36)	37.7 (3.08)	33.3 (1.74)	32.2 (1.23)	35.8 (1.06)	35.9 (0.78)
480	45.6 (2.8)	46.5 (3.86)	40.5 (3.21)	36.2 (1.97)	35.7 (1.28)	39.4 (1.27)	39.9 (0.85)
540	49.6 (3.1)	50 (4.34)	43.3 (3.35)	39.1 (2.19)	39.2 (1.32)	43 (1.47)	43.7 (0.99)
600	53.5 (3.43)	53.3 (4.77)	46.1 (3.44)	42 (2.39)	42.5 (1.42)	46.5 (1.66)	47.3 (1.14)
660	57.2 (3.72)	56.4 (5.07)	48.8 (3.57)	44.8 (2.55)	45.8 (1.53)	49.9 (1.91)	50.6 (1.28)
720	60.8 (4.02)	59.2 (5.31)	51.5 (3.69)	47.6 (2.76)	49 (1.6)	53.1 (2.08)	53.9 (1.46)
780	64.3 (4.27)	61.9 (5.52)	54.1 (3.78)	50.4 (2.95)	51.9 (1.62)	56.1 (2.29)	57 (1.61)
840	67.6 (4.52)	64.6 (5.87)	56.7 (3.86)	53.2 (3.07)	54.8 (1.76)	59.2 (2.45)	59.9 (1.85)
900	70.9 (4.73)	67.2 (6.23)	59.2 (4.04)	55.9 (3.23)	57.6 (1.85)	62.2 (2.58)	62.7 (2.09)
960	74 (4.99)	69.7 (6.49)	61.7 (4.08)	58.5 (3.35)	60.3 (1.94)	65.1 (2.68)	65.3 (2.27)
1020	77 (5.09)	72.1 (6.75)	64.2 (4.13)	61.1 (3.45)	62.9 (2.12)	67.9 (2.74)	67.8 (2.48)

1080	79.8 (5.17)	74.4 (6.85)	66.5 (4.18)	63.6 (3.58)	65.4 (2.31)	70.7 (2.83)	70.2 (2.78)
1140	82.4 (5.13)	76.6 (6.85)	68.8 (4.23)	66 (3.6)	67.9 (2.34)	73.3 (2.95)	72.4 (3)
1200	84.8 (5.06)	78.6 (6.78)	71 (4.29)	68.4 (3.64)	70.2 (2.28)	75.7 (2.99)	74.6 (3.16)
1260	87 (5)	80.4 (6.85)	73.2 (4.34)	70.8 (3.77)	72.5 (2.3)	78 (3.03)	76.6 (3.37)
1320	89 (4.88)	82.1 (6.92)	75.3 (4.32)	73 (3.76)	74.6 (2.25)	80.1 (3.12)	78.4 (3.49)
1380	90.8 (4.72)	83.5 (7.01)	77.4 (4.31)	75.1 (3.83)	76.7 (2.16)	82.2 (3.25)	80.3 (3.57)
1440	92.4 (4.6)	84.9 (7)	79.3 (4.3)	77.1 (3.87)	78.7 (2.07)	83.9 (3.27)	82 (3.73)

Table S4. RSQ results of LOESS models for individual tested tablets.

	RSQs of LOESS models of carvedilol release results for tablet 1 of 4	RSQs of LOESS models of carvedilol release results for tablet 2 of 4	RSQs of LOESS models of carvedilol release results for tablet 3 of 4	RSQs of LOESS models of carvedilol release results for tablet 4 of 4
Polyglykol® 4000 P	0.999038083	0.997826745	0.999247685	0.998265217
Polyglykol® 8000 P	0.998640295	0.998406944	0.9988345	0.998604489
POLYOX™ WSR N-80 (LEO NF Grade)	0.999885176	0.999938454	0.999906771	0.999907963
KOLLIDON® 25	0.999765367	0.998975066	0.999458932	0.998717628
KOLLIDON® 90 F	0.999978743	0.999976363	0.99995287	0.999951725
C*Pharm Mannidex 16700	0.999949674	0.999935552	0.999868613	0.994643682
PEARLITOL® 160C	0.999934256	0.999918568	0.999147116	0.999958689
Parteck® M 100	0.996618012	0.997573979	0.999355216	0.993537404
Parteck® M 200	0.999256112	0.996787743	0.999245382	0.999836075
Lactochem® Fine Powder	0.999871901	0.999985757	0.999953613	0.999845799
Lactochem® Crystals	0.999865964	0.999923965	0.999899913	0.999773615
SuperTab® 11SD	0.999811004	0.999919998	0.999936925	0.999940946
FlowLac® 100	0.999972979	0.999929325	0.99980934	0.999934854
Tablettose® 70	0.999893668	0.999938821	0.999902769	0.999742256
Granulated sugar N°1 600	0.999996352	0.999995209	0.999996771	0.999995688
GLUCIDEX® 19	0.999993076	0.999977473	0.999996493	0.999844563
DI-CAFOS® A12	0.999997634	0.999996272	0.999996698	0.999996746
EMCOMPRESS® Anhydrous	0.999989571	0.999988076	0.999985597	0.999991268
STARCH 1500® ↓PS	0.999989263	0.999986324	0.99994923	0.999986844
STARCH 1500® ↑PS	0.999993686	0.99999496	0.999994487	0.999993311
AVICEL® PH-102	0.999994083	0.999990994	0.999995144	0.999994623
AVICEL® PH-200	0.999992457	0.999998194	0.999995874	0.999998155
ETHOCEL™ Standard 20 Premium	0.999996505	0.999995186	0.999996924	0.99999667
	MIN = 0.993537404; MAX = 0.999998194; MEAN = 0.999546401			

Table S5. LOESS estimated times at which 0 %, 5 %, 10 %,…, 95 % of carvedilol is released. Data is shown for PEG/PEO, PVP and mannitol groups. The results given are mean values with corresponding standard deviations shown in parentheses (n = 4).

$T_{X\%,\ LOESS}$ (min)	Polyglykol® 4000 P	Polyglykol® 8000 P	POLYOX™ WSR N-80 (LEO NF Grade)	KOLLIDON® 25	KOLLIDON® 90 F	C*Pharm Mannidex 16700	PEARLITOL® 160C	Parteck® M 100	Parteck® M 200
$T_{0\%}$, T_{lag}	[a]	1.3 (0.7)	6.7 (0.6)	[a]	3.8 (0.2)	[a]	[a]	6.5 (1.9)	4.2 (2.2)
$T_{5\%}$	[a]	4.5 (0.7)	17.7 (2.1)	[a]	29.4 (5.9)	2.3 (2.4)	[a]	10.2 (0.9)	8.8 (0.6)
$T_{10\%}$	3.9 (0.7)	7.7 (0.8)	28.4 (3.5)	7.3 (2.6)	53.3 (10.4)	11.1 (1.2)	8.4 (1.8)	13.9 (0.8)	13.5 (1.1)
$T_{15\%}$	7.5 (0.6)	10.9 (0.8)	38.1 (3.5)	12 (2.8)	75.1 (14.3)	20 (3.4)	17.9 (2.1)	17.6 (1.8)	18.1 (2.6)
$T_{20\%}$	11.1 (0.7)	14.2 (0.9)	47.2 (3.7)	16.7 (3.4)	96.1 (18.2)	28.9 (6.3)	27.5 (2.5)	21.3 (2.9)	22.8 (4.2)
$T_{25\%}$	14.8 (1.1)	17.5 (0.9)	56.1 (3.7)	21.6 (4.4)	116.4 (21.4)	38.8 (10.2)	37.7 (3.6)	25 (4.1)	27.4 (5.8)
$T_{30\%}$	18.5 (1.5)	20.8 (1)	65.7 (3.2)	26.5 (5.6)	136.5 (24.5)	49.7 (14.7)	47.9 (6)	28.8 (5.4)	31.8 (6.9)
$T_{35\%}$	22.2 (2)	24.1 (1.1)	75.2 (3.2)	32.5 (8)	156.1 (26.9)	61.7 (19.9)	58.7 (9.3)	32.5 (6.5)	36.2 (8.1)
$T_{40\%}$	26 (2.5)	27.4 (1.1)	85.7 (3.6)	40.3 (12.8)	176 (29)	73.9 (25.8)	69.6 (12.9)	36.1 (7.6)	40.8 (9.4)
$T_{45\%}$	29.8 (3)	30.8 (1.2)	95.8 (3.7)	51.4 (21.8)	196.2 (31.4)	86.1 (31.5)	80.3 (17.3)	39.7 (8.8)	45.2 (10.7)
$T_{50\%}$	33.9 (3.9)	34.2 (1.4)	107.1 (5)	64.7 (32.7)	217.2 (33.4)	98.5 (37.5)	91.5 (22.2)	43.4 (10)	51 (13.4)
$T_{55\%}$	38.6 (5.3)	38.4 (1.8)	118.7 (5.7)	79.8 (45.3)	239.4 (35.3)	110.8 (43.8)	103.1 (27.6)	47.5 (11.3)	56.8 (16.2)
$T_{60\%}$	43.6 (6.3)	43 (1.7)	131.5 (7.6)	95.8 (58.9)	263.2 (37.5)	123.3 (50.4)	115.3 (33)	53 (13.3)	63.4 (18.9)
$T_{65\%}$	48.8 (7.1)	47.5 (1.6)	144.8 (8.6)	112.2 (73.2)	289.2 (39.9)	138.3 (53.5)	128.3 (38.9)	58.2 (14.6)	71.7 (22.6)
$T_{70\%}$	56.2 (10.6)	52 (1.6)	159.4 (11.4)	129.2 (87.9)	317.9 (42.1)	153.6 (58.3)	143.8 (44.4)	63.2 (16.1)	79.9 (27.1)
$T_{75\%}$	62.9 (13.2)	57.9 (3)	175.8 (12.9)	147.4 (103.1)	349.4 (43.6)	172.2 (60.1)	159.3 (51.5)	71.4 (20.8)	89.3 (32.9)
$T_{80\%}$	74.4 (18.9)	67 (1.1)	193 (15.4)	169.1 (118.2)	383 (45.7)	192.1 (62.8)	177.8 (60)	80.3 (21)	101.8 (39.5)
$T_{85\%}$	88.7 (25.1)	74.3 (0.6)	213.6 (17)	191.8 (136.8)	422.8 (47.1)	217 (65.9)	200.4 (68)	93.8 (24)	117.7 (46.1)
$T_{90\%}$	122.8 (50.3)	91.2 (1.7)	237.5 (19)	224.1 (155.3)	471.1 (51.1)	248.1 (71.4)	227.2 (80.5)	110.6 (29.6)	136.2 (54.6)
$T_{95\%}$	195.2 (105.3)	114.6 (11.6)	272.2 (20.9)	266.9 (181.7)	534.9 (60.1)	289.6 (82.5)	262.1 (94.3)	135.4 (35.8)	162.5 (60.2)

[a] an ongoing burst release stage for at least one of the tested tablets (n = 4).

Table S6. LOESS estimated times at which 0 %, 5 %, 10 %,…, 95 % of carvedilol is released. Data is shown for the lactose group, sucrose and maltodextrin. The results given are mean values with corresponding standard deviations shown in parentheses (n = 4).

$T_{X\%, LOESS}$ (min)	Lactochem® Fine Powder	Lactochem® Crystals	SuperTab® 11SD	FlowLac® 100	Tablettose® 70	Granulated sugar N°1 600	GLUCIDEX® 19
$T_{0\%}$, T_{lag}	[a]	[a]	[a]	[a]	[a]	[a]	[a]
$T_{5\%}$	[a]	[a]	[a]	[a]	[a]	18.4 (2)	17.3 (4.5)
$T_{10\%}$	19.4 (3.4)	[a]	[a]	14.4 (7.1)	[a]	47.6 (3.3)	50.6 (6)
$T_{15\%}$	38.8 (4.4)	[a]	[a]	36.5 (9.5)	[a]	80.8 (5)	87.6 (7.1)
$T_{20\%}$	62.4 (6.7)	[a]	[a]	64.7 (13.6)	[a]	115.7 (6.5)	127 (9)
$T_{25\%}$	91.2 (8.9)	17.6 (6.5)	[a]	97.4 (16)	[a]	152.4 (8.2)	168.7 (10.3)
$T_{30\%}$	123.4 (12)	31.9 (7.4)	[a]	133.6 (17.1)	17 (6.3)	191.4 (9.6)	211.5 (11.4)
$T_{35\%}$	159.2 (14.7)	50.8 (10.2)	24.9 (16.3)	172.3 (17.9)	29.6 (5.9)	232.9 (11.1)	254.8 (12.5)
$T_{40\%}$	197.1 (16.8)	75 (12.7)	45.1 (20.6)	213.5 (18.9)	46.8 (6.7)	276.6 (12.9)	299.4 (13.4)
$T_{45\%}$	236.5 (17.5)	103.3 (14.4)	69 (25.7)	256.5 (19.8)	70.3 (7.1)	322.4 (14.7)	345 (14.5)
$T_{50\%}$	278 (17.6)	135.8 (16.5)	98.3 (28.1)	301 (20.7)	98.6 (6.4)	371.6 (17.5)	392.4 (15.7)
$T_{55\%}$	322.1 (18)	172.5 (18.4)	133.9 (31.1)	348 (21.5)	132.9 (5.2)	424 (20)	437.8 (22.5)
$T_{60\%}$	369.9 (19)	213.8 (20.2)	176.5 (34.2)	397.7 (22.7)	172.7 (4.8)	479.6 (23.4)	482.3 (31.3)
$T_{65\%}$	420.4 (20.4)	260 (22.5)	223.9 (37.3)	449.5 (23.7)	218.6 (6.8)	539.2 (27.2)	528.2 (40.2)
$T_{70\%}$	473 (22.1)	310.6 (24.8)	276.6 (37.5)	503.2 (24)	270.1 (8.5)	603.9 (32.8)	577.2 (48.8)
$T_{75\%}$	524.9 (26.7)	366.1 (28.3)	335.4 (37.1)	554.4 (28.3)	326.2 (10.7)	677.1 (41)	632 (59)
$T_{80\%}$	576.1 (39.6)	425.7 (32.2)	400.4 (38.1)	602.2 (33.1)	387.7 (10.5)	762.9 (51.7)	698.6 (65)
$T_{85\%}$	625.8 (57.9)	490.6 (35.3)	468 (41.6)	646.7 (38.4)	451.7 (14.9)	875.4 (70.6)	780 (74.2)
$T_{90\%}$	674.5 (75.1)	557 (34.2)	534.9 (45.4)	692.2 (41.9)	516.6 (29.5)	1057.7 (113.9)	883 (89.2)
$T_{95\%}$	724.5 (90.2)	619.3 (30.6)	607.8 (49.9)	1238.4 (929.5)	605 (60.2)	> 1440	1024.3 (117.9)

[a] an ongoing burst release stage for at least one of the tested tablets (n = 4).

Table S7. LOESS estimated times at which 0 %, 5 %, 10 %,…, 95 % of carvedilol is released. Data is shown for DCP, pregelatinized starch, MCC and EC. The results given are mean values with corresponding standard deviations shown in parentheses (n = 4).

$T_{X\%, LOESS}$ (min)	DI-CAFOS® A12	EMCOMPRESS® Anhydrous	STARCH 1500® ↓PS	STARCH 1500® ↑PS	AVICEL® PH-102	AVICEL® PH-200	ETHOCEL™ Standard 20 Premium
$T_{0\%}$, T_{lag}	[a]	[a]	[a]	[a]	[a]	[a]	[a]
$T_{5\%}$	16.8 (0.9)	[a]	[a]	[a]	31.7 (6.7)	28.6 (3.3)	32.7 (2.7)
$T_{10\%}$	52.4 (3.1)	24.9 (2.9)	[a]	40.2 (5.2)	82.1 (12.6)	72.9 (7.8)	84.1 (6.4)
$T_{15\%}$	99.2 (6.4)	60.9 (5.9)	48.7 (22.9)	96.8 (8.7)	145.4 (16.5)	127 (10.3)	140.9 (8.6)
$T_{20\%}$	152.1 (10.5)	111.6 (9.8)	104.7 (37.5)	169.9 (13.3)	220.1 (18.5)	189.2 (11.7)	201.7 (10.3)
$T_{25\%}$	209.5 (15.3)	170.6 (16.1)	179.9 (47.4)	257.3 (21.4)	301.1 (19.4)	257.8 (12.5)	266.4 (11.2)
$T_{30\%}$	270.7 (20.8)	235.8 (23.7)	268.1 (55.2)	355.2 (30.2)	383.9 (20)	331.7 (14)	334.3 (11.4)
$T_{35\%}$	335.5 (26.7)	305.9 (32.5)	367.3 (61.5)	457.6 (39.2)	468.3 (21.6)	409.4 (16.8)	406.7 (12)
$T_{40\%}$	403.8 (33.3)	381.7 (43)	472.3 (68.1)	561.7 (48.6)	555.8 (23.9)	490.8 (21.8)	482.8 (13.9)
$T_{45\%}$	475.2 (40.4)	463.2 (56.2)	579.9 (74.5)	667.8 (57.2)	646.1 (27.2)	576 (27.7)	563.8 (17.6)
$T_{50\%}$	549.8 (48.2)	550.7 (72.5)	690.1 (81.5)	774.9 (65.4)	742.3 (31.7)	665.5 (34.9)	650.7 (22.2)
$T_{55\%}$	628.8 (57.2)	646.4 (92.4)	804.2 (89.7)	884.5 (74.3)	846.3 (37.8)	760.3 (43)	744.1 (28.1)
$T_{60\%}$	711.9 (67)	750.5 (114.8)	922.2 (97)	998.5 (83)	956.8 (46.2)	858.9 (50.3)	845.6 (37)
$T_{65\%}$	799.4 (77.8)	862.8 (132.8)	1045.5 (105.5)	1117.9 (89.9)	1072.9 (53.7)	960.8 (57.2)	957.6 (48.9)
$T_{70\%}$	892.1 (90.5)	982 (147.2)	1176.9 (115.7)	1245.1 (98.4)	1195.5 (59.3)	1069.1 (65.9)	1082.6 (64)
$T_{75\%}$	989.1 (104.6)	1116.1 (171.5)	1314.9 (123.4)	> 1440	1331 (63.8)	1187.3 (79.5)	1222.4 (84.5)
$T_{80\%}$	1093.6 (121.5)	> 1440	> 1440	> 1440	> 1440	> 1440	> 1440
$T_{85\%}$	1214 (141.6)	> 1440	> 1440	> 1440	> 1440	> 1440	> 1440
$T_{90\%}$	> 1440	> 1440	> 1440	> 1440	> 1440	> 1440	> 1440
$T_{95\%}$	> 1440	> 1440	> 1440	> 1440	> 1440	> 1440	> 1440

[a] an ongoing burst release stage for at least one of the tested tablets (n = 4).

Table S8. Observed similarity factors (f_2) and bootstrapped (n = 5000) lower bound 90 % confidence interval estimations of similarity factors (shown in parentheses) for carvedilol release from METHOCEL™ K15M Premium matrix tablets containing different selected excipients as carvedilol release modifiers. Data is separately shown for PEG/PEO, PVP and mannitol groups (a), lactose group, sucrose and maltodextrin (b) and finally for all of the insoluble selected excipients (c). Green colouration denominates 'similar' carvedilol release profiles according to the f_2 metric, orange colouration denominates 'partial similarity' of carvedilol release profiles (only observed f_2 is above 50.0).

a)

	PEG 8k	PEO	PVP K25	PVP K90	MAN_C_1	MAN_C_2	MAN_SD_1	MAN_SD_2
PEG 4k	81.5 (70.3)	36.9 (34.5)	53.3 (41)	25.5 (23.6)	41.7 (35.7)	41.2 (37)	57.7 (52.4)	58.1 (49.6)
PEG 8k	/	36.1 (35.2)	51.5 (38.3)	24.7 (23.3)	40.5 (35.2)	40.5 (36.6)	61.5 (54.4)	59.2 (49.5)
PEO	/	/	45.7 (36.8)	38 (34.8)	63.9 (51.1)	56.8 (48.2)	39 (35.3)	43.4 (37.6)
PVP K25	/	/	/	30.9 (25.1)	55.9 (41)	56.2 (43)	54.9 (40.8)	61.2 (44.3)
PVP K90	/	/	/	/	36.3 (30.3)	33.9 (29)	25.4 (23.3)	27.5 (24.6)
MAN_C_1	/	/	/	/	/	69.2 (50.5)	43.8 (36.4)	49.1 (39.2)
MAN_C_2	/	/	/	/	/	/	45.7 (38.6)	50.4 (41.2)
MAN_SD_1	/	/	/	/	/	/	/	73 (55)

b)

	LAC_M	LAC_C	LAC_SD_1	LAC_SD_2	LAC_AG	SUC	MD_SD
PEG 4k	24.1 (22.9)	33.4 (31.4)	36.5 (33.9)	23.5 (22.3)	36.9 (34.9)	20.2 (19.2)	19.8 (18.8)
PEG 8k	23.3 (22.8)	32 (30.9)	34.7 (33)	22.7 (22)	35.1 (34.6)	19.4 (18.9)	19 (18.6)
PEO	31.9 (30.8)	41.5 (40.2)	41.5 (40.7)	30.6 (29.5)	42.7 (41.7)	26.1 (25.3)	25.7 (24.9)
PVP K25	28.4 (23)	40.4 (31.9)	43.5 (34.9)	27.3 (22.4)	43.7 (34.1)	22.7 (18.8)	22.6 (18.7)
PVP K90	51.1 (46.6)	46.1 (43.2)	40.8 (37.6)	48.1 (44.2)	41.3 (38.9)	38.7 (36.1)	39.3 (36.2)
MAN_C_1	31.4 (27.7)	43.8 (37.7)	45.3 (40.1)	30.1 (26.3)	46.3 (40.7)	25.1 (22.2)	24.9 (22)
MAN_C_2	29.5 (26.1)	40.4 (35.4)	41.7 (36.8)	28.1 (24.9)	41.9 (37)	23.3 (20.8)	23.3 (20.7)
MAN_SD_1	23.3 (22.1)	31.8 (30)	33.9 (31.9)	22.5 (21.3)	34.2 (32.4)	19 (18)	18.8 (17.8)
MAN_SD_2	25.1 (23.3)	34.5 (31.8)	36.8 (33.8)	24.3 (22.5)	37.2 (34.6)	20.6 (19.1)	20.3 (18.9)
LAC_M	/	45.4 (42.8)	40.1 (37.3)	77.6 (68.2)	39.9 (38.8)	47.7 (44.5)	51.1 (47)
LAC_C	/	/	70.1 (60.4)	43.3 (40.7)	67.1 (61.6)	33.8 (32.3)	34.1 (32.4)
LAC_SD_1	/	/	/	38.8 (35.9)	83.6 (73.4)	31.3 (29.4)	31.3 (29.3)

LAC_SD_2	/	/	/	/	38.7 (37.3)	52.4 (48.5)	55.6 (50.7)
LAC_AG	/	/	/	/	/	31.6 (30.8)	31.4 (30.5)
SUC	/	/	/	/	/	/	69.1 (61.2)

c)

	DCP_1	DCP_2	PS_1	PS_2	MCC 101	MCC 200	EC
PEG 4k	16.7 (15.8)	17.3 (16.2)	15.9 (14.8)	14.3 (13.6)	13.7 (13.1)	14.8 (14.1)	14.7 (14)
PEG 8k	16.1 (15.4)	16.6 (15.7)	15.2 (14.3)	13.7 (13.1)	13.2 (12.7)	14.2 (13.8)	14.1 (13.7)
PEO	20.8 (19.9)	21 (19.8)	18.9 (17.8)	17.2 (16.5)	16.8 (16.3)	18.3 (17.7)	18.2 (17.7)
PVP K25	18.3 (15.4)	18.5 (15.7)	16.7 (14.3)	15 (12.8)	14.6 (12.5)	15.9 (13.5)	15.8 (13.4)
PVP K90	28.7 (26.7)	28.1 (25.9)	24.4 (22.8)	22.5 (21.3)	22.5 (21.5)	24.6 (23.4)	24.6 (23.5)
MAN_C_1	20 (17.8)	20.2 (17.9)	18.1 (16.1)	16.4 (14.8)	16 (14.3)	17.4 (15.6)	17.3 (15.5)
MAN_C_2	18.4 (16.4)	18.5 (16.5)	16.5 (14.7)	14.9 (13.4)	14.6 (13.1)	16 (14.3)	15.8 (14.2)
MAN_SD_1	15.4 (14.5)	15.8 (14.7)	14.3 (13.3)	12.8 (12.1)	12.4 (11.8)	13.5 (12.8)	13.3 (12.7)
MAN_SD_2	16.7 (15.5)	17.1 (15.8)	15.5 (14.3)	14 (13.1)	13.5 (12.7)	14.7 (13.8)	14.5 (13.7)
LAC_M	33.8 (31.4)	32.5 (29.7)	27.9 (26.1)	25.6 (24.3)	25.7 (24.6)	28.3 (27)	28.2 (27)
LAC_C	26.2 (24.7)	26.1 (24.2)	23.1 (21.6)	20.9 (20)	20.6 (19.9)	22.5 (21.7)	22.4 (21.6)
LAC_SD_1	24.8 (23.2)	25 (23.1)	22.3 (20.7)	20.2 (19)	19.8 (18.7)	21.5 (20.4)	21.3 (20.2)
LAC_SD_2	36.2 (33.7)	34.9 (31.9)	29.8 (27.9)	27.3 (26)	27.4 (26.4)	30.3 (28.9)	30.1 (28.9)
LAC_AG	25.1 (24)	25.4 (23.9)	22.8 (21.4)	20.6 (19.8)	20.1 (19.6)	21.9 (21.3)	21.7 (21.2)
SUC	49.2 (44.6)	46.1 (41)	38 (35.2)	34.8 (32.7)	35.2 (33.4)	39.5 (37.3)	39.3 (37.1)
MD_SD	44.8 (40)	41.2 (36.5)	34.5 (31.7)	31.9 (29.5)	32.5 (30.3)	36.3 (33.6)	36 (33.6)
DCP_1	/	69.2 (57.8)	53.2 (47.2)	48.7 (43.6)	49.9 (45.1)	59.7 (52.3)	58.4 (51.7)
DCP_2	/	/	62.8 (51.5)	53.6 (46.1)	52.9 (46.1)	62.4 (53.3)	61.1 (52.7)
PS_1	/	/	/	70.1 (58.7)	62.9 (55.8)	65.4 (60.5)	64.3 (59.5)
PS_2	/	/	/	/	78.8 (72)	65.9 (58.2)	66 (58.8)
MCC 101	/	/	/	/	/	70.9 (63.5)	72.3 (64.7)
MCC 200	/	/	/	/	/	/	93.4 (79.4)

Table S9. Particle size distribution results obtained using the laser diffraction method. The results of particle size distribution are given based on volume-weight diameter (D_{10}, D_{50}, D_{90}, mean-$D_{(4,3)}$).

Ingredient name	$D_{(4,3)}$ (µm)	D_{10} (µm)	D_{50} (µm)	D_{90} (µm)
METHOCEL™ K15M Premium	92	27	75	181
Carvedilol (free base)	37	8	30	75
Magnesium stearate EUR PHAR	5	2	4	10
Polyglykol® 4000 P	340	40	327	642
Polyglykol® 8000 P	305	47	262	625
POLYOX™ WSR N-80	227	63	192	441
KOLLIDON® 25	68	20	60	128
KOLLIDON® 90 F	208	85	185	363
C*Pharm Mannidex 16700	72	9	48	172
PEARLITOL® 160C	88	8	52	224
Parteck® M 100	57	13	51	108
Parteck® M 200	114	20	106	216
Lactochem® Crystals	146	28	138	271
Lactochem® Fine Powder	54	6	35	120
SuperTab® 11SD	110	38	100	195
FlowLac® 100	106	27	94	201
Tablettose® 70	75	13	57	164
Granulated sugar N°1 600	707	326	667	1175
GLUCIDEX® 19	130	13	112	278
DI-CAFOS® A12	12	1	10	26
EMCOMPRESS® Anhydrous	191	60	186	322
AVICEL® PH-102	126	34	113	238
AVICEL® PH-200	199	56	186	356
ETHOCEL™ Standard 20 Premium	372	124	326	694
STARCH 1500® QbD sample with smaller particle size (↓PS)	70	13	56	154
STARCH 1500® QbD sample with larger particle size (↑PS)	94	16	86	185

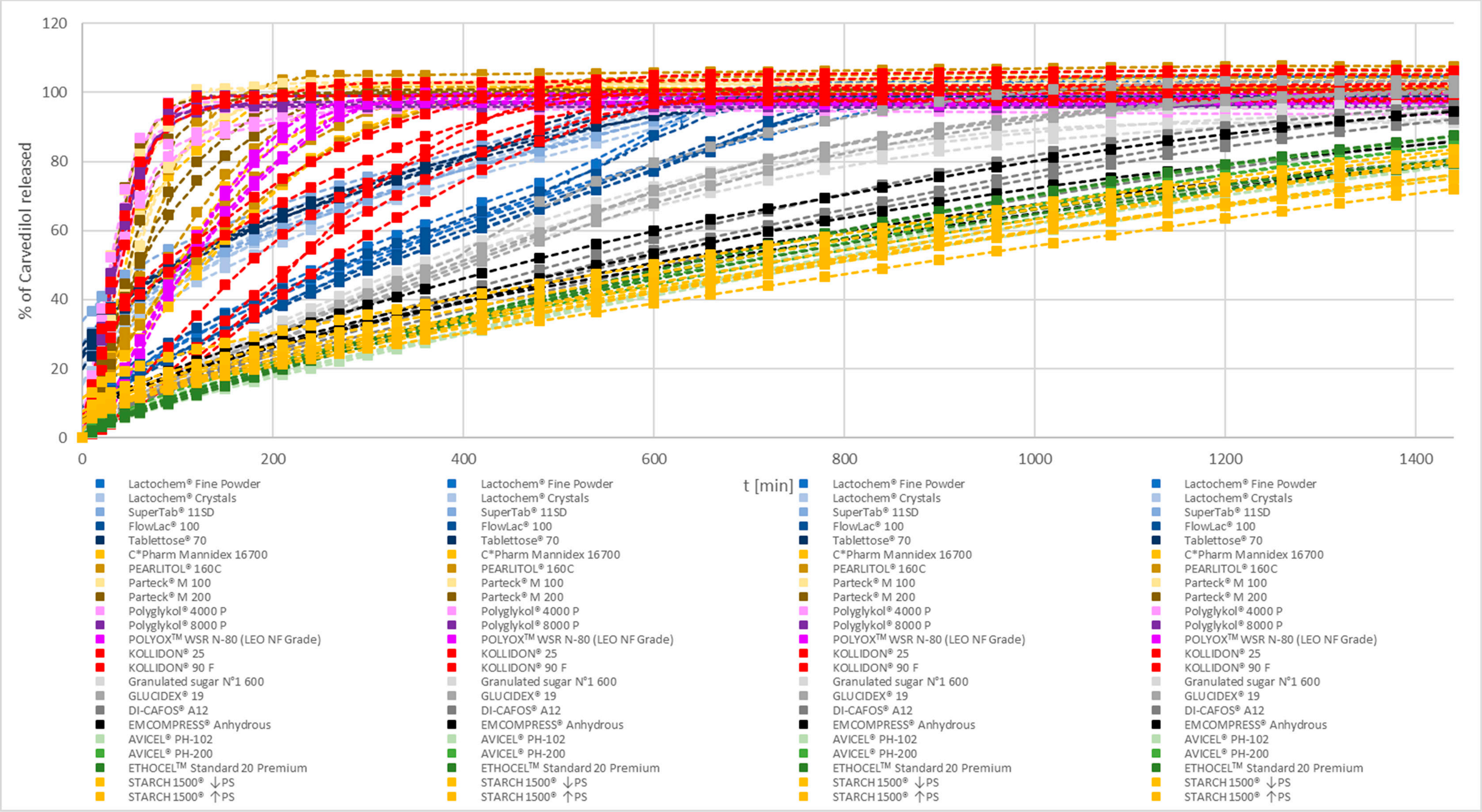

Figure S1. Comparison of carvedilol release from METHOCEL™ K15M Premium matrix tablets containing different selected excipients as carvedilol release modifiers. Points in the graph represent experimentally measured carvedilol release for individual tested tablets and dashed lines represent LOESS models with 1 min resolution.
